# Case of a Stone of the Bladder

**Published:** 1784

**Authors:** 


### SECTION II.

### Essays and Observations.

I. *Caſe of a Stone of the Bladder. Communicated in a letter to Dr.* Simmons, F.R.S. *by* Benjamin Chandler, M.D. *Phyſician at Canterbury, and Extra-Licentiate of the Royal College of Phyſicians, London.*

THE following particulars concerning a patient of mine, Mr. Charles Noble, who died the other day in this city, are very much at your ſervice, if you think they merit a place in the London Medical Journal. His body was opened in my preſence, and the largeſt ſtone taken out of his bladder, that I can recollect ever to have ſeen or heard of. It filled the whole pelvis, and the bladder was ſo thickened in its coats, and its veſſels ſo diſtended, that at firſt ſight it reſembled a uterus. The ſtone, which was no where attached, was of a depreſſed oval figure, with the ſmalleſt end downwards, tolerably regular in its ſhape, very compact, and weighed ſeventeen ounces, averdupoiſe. It meaſures twelve inches in circumference; is two inches thick at its broadeſt end, and two and a half at the narroweſt, towards the neck of the bladder. From the ſmaller extremity was broken off a ſmall fragment, which had partly ſunk into the beginning of the urethra, and with a quantity of gritty ſand, which ſeemed to have been detached with it, would have weighed about half an ounce more, if we had been curious in collecting it.

Mr. Noble began to feel ſymptoms of the ſtone about twenty years ſince, and being a man of uncommon reſolution, took, in ſucceſſion, all the lithontriptics, that have acquired any character, with the utmoſt perſeverance.

During

During the laſt five years of his life, however, every medicine of that ſort had been laid aſide, and my ſole object was to keep him as eaſy as, under ſuch circumſtances, he could be ; and in this I ſucceeded ſo far, by means of a very abſtemious and bland diet, and by keeping his bowels lax, as to enable him to enjoy the company of his friends over a game of quadrille every evening, during the winter, and to walk about our rugged ſtreets this ſummer.

For ſeveral weeks before he died, he was in conſtant pain, notwithſtanding a liberal uſe of opiates, and I conjecture that the breaking off of the fragment, was the cauſe that I could never get him eaſy again, as I had done before. But I believe the immediate cauſe of his death was the burſting of a vomica ; the foundation of which was laid in a *pneumonia* laſt autumn.

*Canterbury,*
*September* 16, 1784.

